# Correlation between glucose metabolism parameters derived from FDG and tumor TNM stages and metastasis-associated proteins in colorectal carcinoma patients

**DOI:** 10.1186/s12885-021-07944-z

**Published:** 2021-03-09

**Authors:** Mingyu Zhang, Jigang Yang, Hao Jiang, Huijie Jiang, Zhenchang Wang

**Affiliations:** 1 Department of Nuclear Medicine, Beijing Friendship Hospital, Capital Medical University, No.95 Yongan Road, Xicheng District, Beijing, China; 2 Department of Radiology, Second Affiliated Hospital of Harbin Medical University, No. 246 Xuefu Road, Nangang District, Harbin, Heilongjiang Province China; 3 Department of Radiology, Beijing Friendship Hospital, Capital Medical University, No. 95 Yongan Road, Xicheng District, Beijing, China

**Keywords:** Positron emission tomography, Colorectal carcinoma, Fluorodeoxyglucose, Glucose transporter 1, Metastasis-associated in colon cancer 1

## Abstract

**Background:**

The aim of this study was to investigate the relationship between multiple metabolism parameters derived from FDG and tumor TNM stages as well as tumor metastasis-associated protein of GLUT-1 and MACC1 in colorectal carcinoma (CRC).

**Methods:**

Thirty-eight patients (24 males and 14 females) with primary CRC confirmed by elective surgery pathological, who also accepted ^18^F-FDG PET/CT scans during 2017 to 2019 were included in this study. The tumor classification of T, N and M is explained by the 7th American Joint Committee on Cancer (AJCC). ^18^F-FDG parameters of SUVmax, SUVmean, TLG and MTV were measured by drawing a region of interest on the primary lesions. The expression of GLUT-1 and MACC1 was quantified by immunohistochemical, and the correlation between metabolism parameters and tumor biomarkers were analyzed.

**Results:**

According to our analysis, the ^18^F-FDG parameters of SUVmean was significantly correlated with tumor M status (*P* = 0.000) of primary CRC. The primary tumor lesion with higher SUVmax, TLG and MTV values prone to a high-T status (*P* = 0.002, 0.002 and 0.001, respectively). The high expression of GLUT-1/MACC1 weas more frequently involved with T3–4 stage and was poorly differentiated in CRC patients. Multivariate analysis found that the expression of GLUT-1 protein was correlated with SUVmax and MTV (*R*^*2*^ = 0.42, *P* = 0.013 and 0.004, respectively), moreover, the expression of MACC1 protein was correlated with TLG (*R*^*2*^ = 0.372, *P* = 0.000).

**Conclusion:**

Glucose metabolism parameters derived from FDG provides a noninvasive assessment of M status and T status in CRC patients. The expression of GLUT-1 and MACC1 was associated with ^18^F-FDG uptake in CRC patients.

## Background

Colorectal carcinoma (CRC) is the third most commonly diagnosed malignancy and the fourth cause of cancer-related death worldwide [1, 2]. Recently, cancer registry data shows adults who are younger than 50 get the higher chance to be suffered from the CRC in some high-income countries [3]. Changes in dietary patterns which is called as western lifestyle are considered to be high risk factors for CRC. Early evaluation of tumor status contributes to the development of therapeutic management and prognostic prediction.

Glucose transporter-1 (GLUT-1) is a member of the membrane transport protein within the glucose transporter family. It has been found to be overexpressed in variety of malignancies including CRC, and expression level of GLUT-1 was correlated with tumor metastatic potential and poor prognosis [4, 5]. In previous studies, higher expression of GLUT-1 was considered as an independent prognostic indicators of worse outcome in T1–2 stage CRC [6]. MACC1 is another promising biomarker in early diagnosis and predict metastasis potential of CRC [7]. Accumulating evidence of in vivo and in vitro studies indicated that high expression of MACC1 was strongly associated with tumor formation, metastases development and poor prognosis [8, 9]. Therefore, GLUT-1 and MACC1 may established as a suitable biomarker for predicting the tumor TNM stage of CRC patients, in order to improve individualized therapy regimen in CRC.

Conventional evaluation of tumor staging relies on electronic colonoscopy, multiple sites CT and MRI scanning and immunohistochemistry evaluation under biopsy and it is difficult to reflect the overall appearance of the tumor in such way. The complexity of the examination was also increased. The integration of various technologies to obtain optimal diagnostic approach is necessary for clinical treatment. Clinical application of ^18^F-FDG PET/CT was considered to be an important method to diagnosis, staging and monitor the therapeutic response of CRC [10, 11]. The parameters of ^18^F-FDG reflected the tracer uptake by tumor cells and provides objective data on tumor glucose metabolism. Recently, the correlation between radio-parameters derived from FDG-PET and tumor biomarkers has been extensively investigated, especially GLUT-1, high expression of GLUT-1 is identified as the promotion of the FDG uptake in wide variety of solid tumors [12]. However, the role of MACC1 in contribute to FDG uptake needs further exploration.

In this study, we retrospectively assessed the feasibility of multiple radio-parameters derived from FDG in predicting tumor stages as well as determine the correlation between GLUT-1/MACC1 and TNM stage and tumor glucose metabolism of CRC. Our findings may provide a useful image data in preoperative evaluation of CRC status.

## Methods

### Patient selection

We retrospectively analyzed 38 patients (24males and 14females) histologically diagnosed with CRC, who accepted surgery excision during January 2017 to July 2019, who also underwent ^**18**^**F-**FDG PET/CT scan prior to surgery. Inclusion criteria: 1) All patients were diagnosed with primary CRC for the first time without any treatment; 2) Patient has complete clinical, pathological and imaging data; 3) Patient did not merge with other parts of the primary tumor; 4) Patient performs PET / CT scan for the first time. Clinicopathological data including sex, age, T status, N status, M status, Differentiation status were obtained. Primary lesion specimens were obtained for immunohistochemical analysis.

### ^18^F-FDG PET/CT protocol and imaging analysis

All patients required fasting for more than 6 h before PET/CT examination and blood glucose levels were measured by a dedicated electronic glucometer to controlled levels of < 10.0 mmol/L. The whole-body PET/CT was conducted with a three-dimensional mode using a PET-CT scanner (Biograph 64 HR, Siemens Medical Solutions). ^18^F-FDG (approximately 259–370 MBq) was injected intravenously 1 h-post the scanning. Patients in supine position with the arms up were scanned. Firstly, CT scan were performed to (120 kV, 200 mA and slice thickness of 3 mm) attenuation correction and anatomical localization, and the PET scan was performed in a three-dimensional mode with a 2.5 min per bed position and generally scans 6-8beds. PET attenuation correction was based on CT data and PET images were reconstructed using an iterative algorithm.

For FDG semi-quantitative analysis, a three-dimensional region of interest (ROI) was manually drawn on the primary lesion and analyzed on Advantage Workstation. The maximum/mean standard uptake value (SUVmax/SUVmean), metabolic tumor volume (MTV), and total lesion glycolysis (TLG) of the primary CRC lesions were then obtained and recorded. A maximal pixel value of 40% was used for ROI measurements.

### Immunohistochemical analysis

Paraffin-embedded CRC specimens were cut into 4 mm-thick sections and immunohistochemical staining was performed as previously described [13]. Briefly, the slides were incubated with anti-GLUT-1 (1:100, Abcam) or anti-MACC1 (1:80, Abcam) at 4 °C overnight. Next, the slides were incubated with horseradish peroxidase-labeled secondary antibody (Boster, Wuhan, China) at room temperature followed by counterstaining with hematoxylin. The staining was observed under a BX53 Olympus microscope (Olympus, Japan) at magnification 200×. A brown-yellow staining was defined as positive expression. Quantitation of GLUT-1 or MACC1 protein were calculated by three different fields of vision randomly per slice via the use of Image-J software (NIH, Bethesda, MD, USA). The IHC optical density score was measured as previous study [14].

### Statistical analysis

All data was expressed as mean ± standard deviation. Statistical analyses were performed with SPSS version 19.0 (IBM, Armonk, NY, USA). Multiple stepwise regression test was used to evaluate the relationship between tumor markers or radio-parameters and clinicopathologic parameters, as well as the relationship between radio-parameters and tumor markers. The receiver operating characteristic (ROC) curve was performed to evaluate the diagnostic accuracy of SUVmax, SUVmean, TLG or MTV cut-off values that best predicted tumor T status or M status. A *P* value < 0.05 was considered statistically significant.

## Results

### Patient population

Totally, 38 patients accepted whole-body ^18^F-FDG PET/CT before surgery during 2017 to 2019 were included in this study. The diagnosis of primary CRC lesions was confirmed by pathology. The majority were male (24, 63.2%) and the average age was 63.79. Twenty-eight (73.7%) patients with T3 stage. Four patients with T4 stage (10.5%). Only six patients with T1 and T2 stage (15.8%). Fifteen (39.5%) patients were existed synchronous distant metastasis at time of diagnosis. The majority of pathologic subtype was moderately differentiated (76.3%), followed by poorly differentiated (18.4%) and well differentiated (5.3%). Thirteen (34.2%) patients had on lymph metastasis, moreover 25 (65.8%) patients had lymph metastasis (AJCC 7th). Details of patients’ characteristics are summarized in Table 1.

**Table 1:** Patient and tumor characteristics

Characteristic	No. of patients	Value (%)
**Sex, male/female**	38(24/14)	63.2/36.8
**Age (years)**		
Mean ± SD		63.79 ± 13.94
Range		32–89
**T stages**		
T1/T2	6	15.8
T3	28	73.7
T4	4	10.5
**N stages**		
N0	13	34.2
N1	13	34.2
N2	12	31.6
**M stages**		
M0	23	60.5
M1	15	39.5
**Differentiation**		
Well	2	5.3
Moderate	29	76.3
Poorly	7	18.4

The clinical stages shown in this table were categorized according to the AJCC (7th guideline)

### Relationship between TNM stages and the expression of GLUT-1 and MACC1 in CRC

GLUT-1 and MACC1 play an important role in tumor progression, we next to investigated the relationship between GLUT-1/MACC1 and tumor T, N and M status in primary CRC lesions. For immunohistochemical staining, the positive expression of GLUT-1 was localized to the membranous of CRC cells, while MACC1 protein was localized to the cytoplasm. The expression of MACC1 protein was higher than GLUT-1 in the same patient. Multivariate analysis shown a significant relationship between GLUT-1/MACC1 expression and T status (*P* = 0.006 and 0.002), also between GLUT-1/MACC1 expression and differentiated degree (*P* = 0.009 and 0.014, Table 2). CRC patients with high T-status and poorly differentiated tended to have higher GLUT-1 and MACC1 expression levels.

**Table 2:** Multivariate regression analysis of the relationship between TNM status and the expression of GLUT-1 and MACC1 of 38 patients with CRC

Variable	No. of patients	GLUT-1	*P* value	MACC1	*P* value
**Sex**	38		0.976		0.217
Male	24	37.87 ± 12.39		58.91 ± 17.03	
Female	14	35.71 ± 9.17		51.22 ± 11.70	
**T status**			0.006		0.002
1/2	6	23.44 ± 4.91		41.42 ± 15.54	
3	28	37.85 ± 9.50		55.98 ± 12.10	
4	4	52.13 ± 4.93		78.76 ± 12.46.	
**N status**			0.072		0.711
N0	13	30.19 ± 6.40		50.82 ± 15.21	
N1	13	37.38 ± 10.46		53.12 ± 15.07	
N2	12	44.20 ± 12.22		64.98 ± 13.63	
**M status**			0.682		0.843
M0	23	34.98 ± 11.60		52.68 ± 15.06	
M1	15	40.29 ± 10.20		61.29 ± 15.36	
**Differentiation**			0.009		0.014
Well	2	22.15 ± 7.64		29.17 ± 8.73	
Moderate	29	35.18 ± 9.27		54.39 ± 11.62	
Poorly	7	49.19 ± 10.27		70.77 ± 18.59	

### Relationship between TNM stages and FDG parameters in CRC

**Fig. 1:**
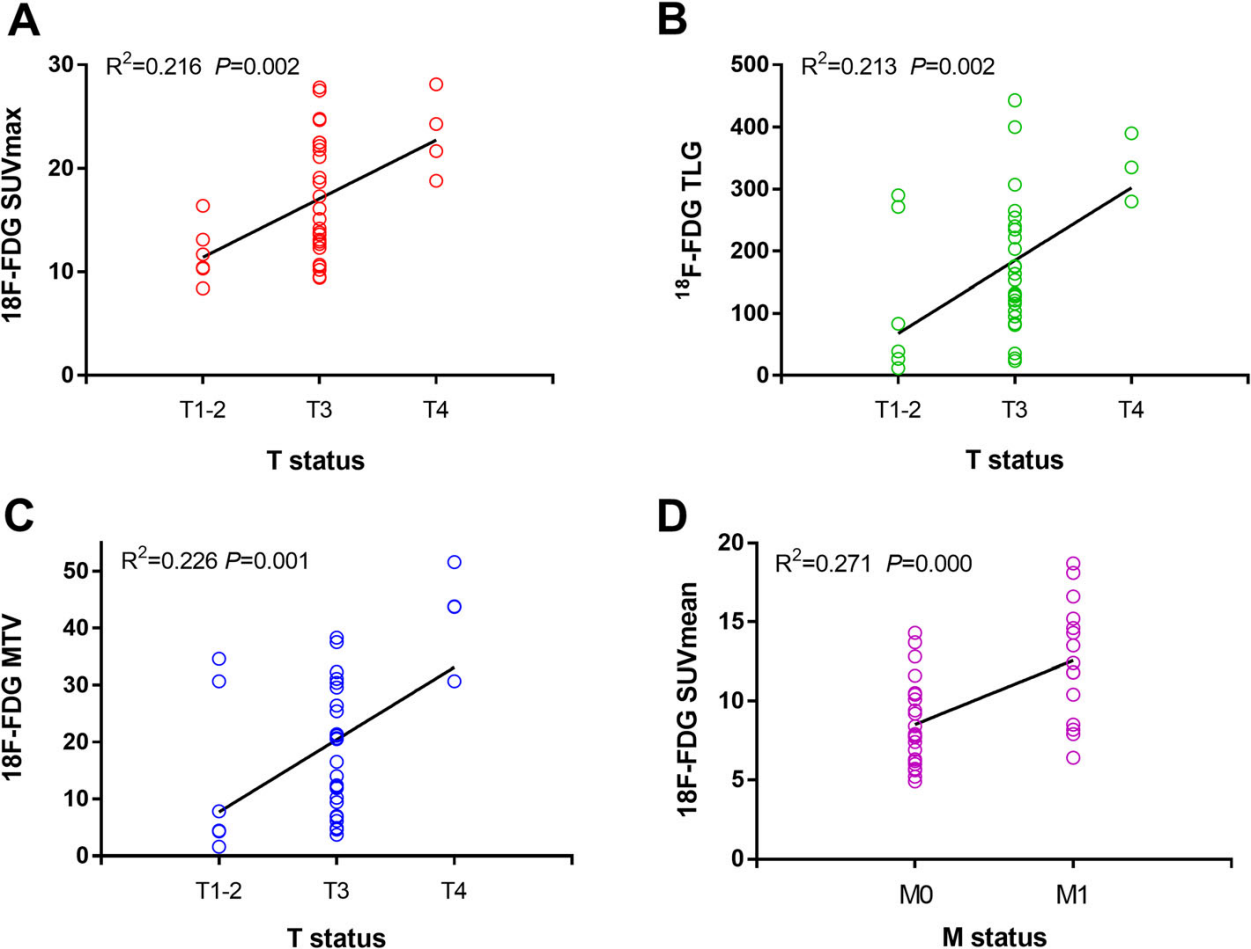
The correlation plot of TNM stages and FDG parameters in CRC. **a** The correlation between SUVmax and T status. **b** The correlation between TLG and T status. **c** The correlation between MTV and T status. **d** The correlation between SUVmean and M status

All the primary lesions of CRC which quantitative by ROI measurements with a SUVmax greater than 2.5 was identified as positive. The SUVmax, SUVmean, TLG and MTV values in patients with different T, N, M or D (differentiation) status were calculated. According to our multivariate analysis, both SUVmax, TLG and MTV values had a statistical correlated with T status (*P* = 0.002, 0.002 and 0.001, respectively), meanwhile SUVmean values had a statistical correlated with M status (*P* = 0.000) of primary CRC (Fig. 1 and Table 3). ROC analysis showed that the area under the curve (AUC) was 0.838 (*P* = 0.029), 0.819 (*P* = 0.01), 0.941 (*P* = 0.04) and 0.96 (*P* = 0.003) for SUVmax, SUVmean, TLG and MTV, respectively. SUVmax cut-off value of 18.75 showed sensitivity of 100%, specificity of 70.6%, MTV cut-off value of 30.5 showed sensitivity of 100%, specificity of 82.4% and TLG cut-off value of 275.85 showed sensitivity of 100%, specificity of 88.2% in distinguish CRC T3 from T4 status. A SUVmean cut-off value of 11.7 showed sensitivity of 66.7%, specificity of 87% in distinguish CRC M status.

**Table 3:** Multivariate analysis of the relationship between T, N and M status and ^18^F-FDG parameters of 38 patients with CRC

Variable	SUVmax		SUVmean		TLG		MTV	
	Mean ± SD	*P* value	Mean ± SD	*P* value	Mean ± SD	*P* value	Mean ± SD	*P* value
**T status**		0.002		0.067		0.002		0.001
1/2	11.72 ± 2.78		7.28 ± 1.54		120.50 ± 126.64		13.90 ± 14.71	
3	16.94 ± 5.58		10.04 ± 3.49		162.51 ± 105.07		17.75 ± 10.42	
4	23.23 ± 3.95		14.88 ± 3.33		381.33 ± 102.50		42.45 ± 8.66	
**N status**		0.212		0.140		0.419		0.647
N0	14.38 ± 5.54		8.49 ± 3.30		139.83 ± 106.78		19.23 ± 12.66	
N1	16.39 ± 5.46		10.27 ± 4.37		160.68 ± 98.61		19.23 ± 12.95	
N2	19.78 ± 5.51		11.69 ± 2.82		240.99 ± 158.89		24.82 ± 13.95	
**M status**		0.244		0.000		0.573		0.836
M0	15.03 ± 5.37		8.51 ± 2.72		163.30 ± 122.55		17.54 ± 11.93	
M1	19.45 ± 5.56		12.56 ± 3.79		202.84 ± 135.50		23.11 ± 15.12	
**Differentiation**		0.112		0.120		0.108		0.612
Well	10.35 ± 0.07		7.10 ± 1.13		55.30 ± 40.30		4.35 ± 0.07	
Moderate	16.13 ± 5.27		9.77 ± 3.72		162.19 ± 107.04		19.39 ± 12.53	
Poorly	21.29 ± 6.22		12.39 ± 3.29		283.49 ± 165.35		25.59 ± 15.81	

### Relationship between GLUT-1/MACC1 expression and ^18^F-FDG uptake in primary CRC lesions

**Fig. 2:**
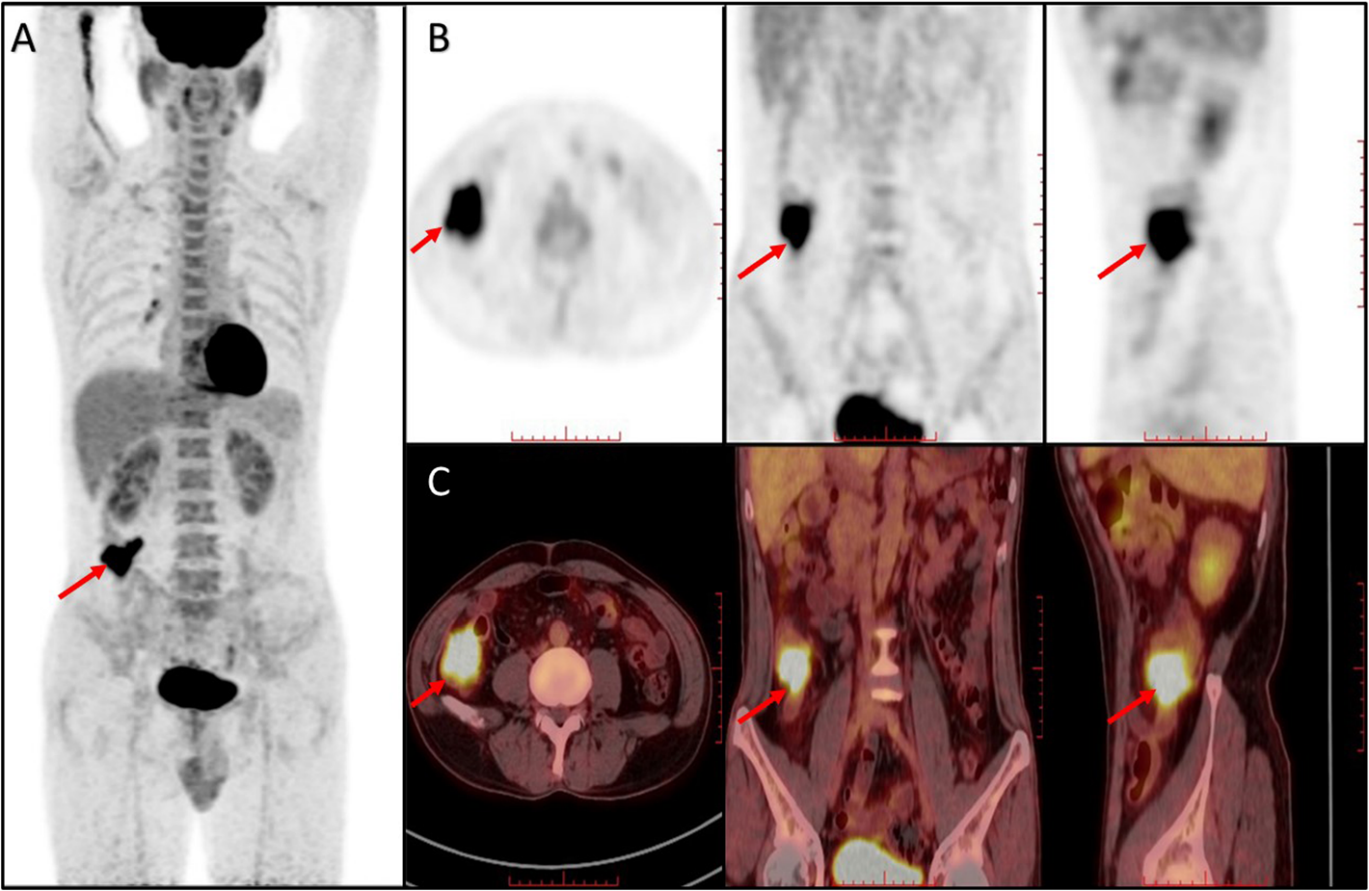
^18^F-FDG PET/CT image of CRC patient. **a** Multiple intensity projection (MIP) of a patient with CRC in T3 stage. **b** The uptake of FDG show in PET image with cross axial, coronary and sagittal position. **c** The uptake of FDG show in PET/CT image with cross axial, coronary and sagittal position. (SUVmax: 9.7, SUVmean: 6.2, TLG: 132.3, MTV: 21.4). Red arrows indicate the uptake of FDG in tumor lesions

**Fig. 3:**
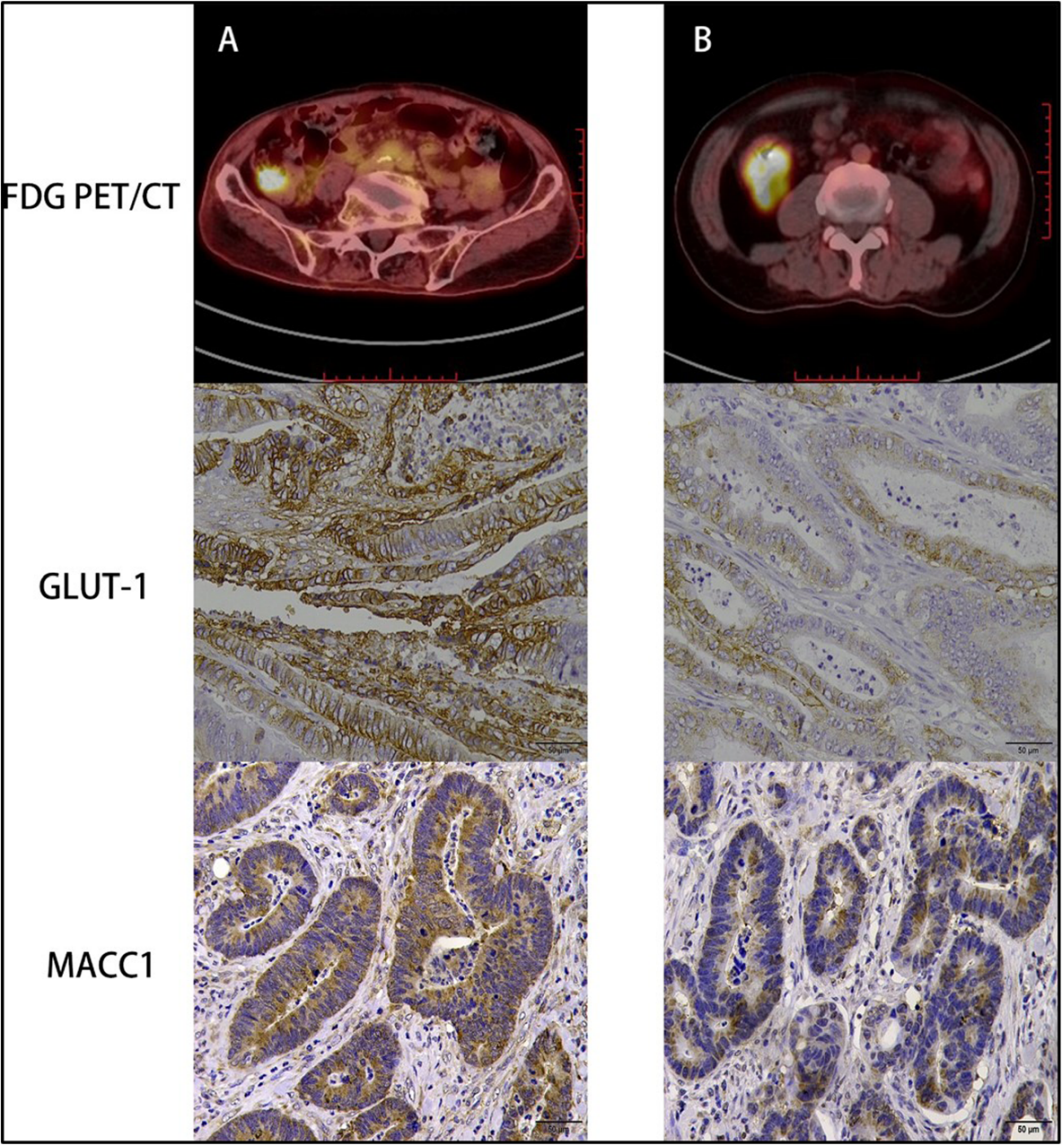
The expression level of GLUT-1 and MACC1 in CRC patients with different TNM stage. **a** A patient with CRC in T3, N2 and M1 (liver metastases) stage, showed a high expression of GLUT-1 and MACC1 in CRC specimen. **b** A patient with CRC in T2, N0 and M0 stage, show a low expression of GLUT-1 and MACC1 in CRC specimen

As GLUT-1 and MACC1 were a promising biomarker to distinguish tumor progression and malignant degree. We investigate whether GLUT-1/MACC1 expression can reflect tumor glycometabolism. Through multivariate analysis the expression of GLUT-1 protein was correlated with SUVmax and MTV (*R*^*2*^ = 0.42, *P* = 0.013 and 0.004, respectively), moreover, the expression of MACC1 protein was correlated with TLG (*R*^*2*^ = 0.372, *P* = 0.000). The expression levels of GLUT-1 in CRC primary lesion may better reflect the character tumor glucose metabolism volume and MACC1 may reflect total lesion glycolysis (Figs. 2 and 3).

## Discussion

Imaging technology is commonly used in the evaluation for the initial diagnosis, staging, restaging and monitoring treatment of CRC, especially those cannot traversed colonoscopically. MRI has a significant advantage in assessing T staging of primary rectal cancer because of the capacity to differentiate the intestinal wall laminar structure [15]. However, evaluation of N and M stage in patients with CRC using conventional procedures was still present challenges until the emergence of whole-body ^18^F-FDG PET/CT. PET/CT integrate morphologic and molecular data to detecting tumor biological behavior, furthermore provide lymph nodes and distant metastases information.

In this study, we detected the semi-quantitative (SUVmax, SUVmean, TLG and MTV) derived from FDG and the expression of tumor markers (GLUT-1 and MACC1) in patients with different stages of CRC which consistent with pathological results. Our finding demonstrated that a significant correlation was found among SUVmax, TLG and MTV values and T status (*P* = 0.002, 0.002 and 0.001, respectively), meanwhile another correlation was found between SUVmean values and M status (*P* = 0.000) of primary CRC. The area under the curve (AUC) of SUVmax, SUVmean, TLG and MTV was 0.838, 0.819, 0.941 and 0.96, respectively. For distinguishing T3 status from T4 of CRC, the SUVmax cut-off value of 18.75 showed sensitivity of 100%, specificity of 70.6%, MTV cut-off value of 30.5 showed sensitivity of 100%, specificity of 82.4% and TLG cut-off value of 275.85 showed sensitivity of 100%, specificity of 88.2%. For distinguishing M status of CRC, SUVmean cut-off value of 11.7 showed sensitivity of 66.7%, specificity of 87%. These results suggested that enhanced glucose uptake is associated with malignant tumor progression, similarly FDG parameters data provide metabolic characteristic that have the capacity to distinguish tumor T stage and M stage of CRC. The same results are consistent with previous study, it is considered that SUV value of FDG PET/CT is more suitable for assessment tumor stage compared with CT and MRI, and the combination of colonoscopy will be more beneficial for CRC staging [16, 17]. Clinically, PET/CT scanning is an essential project to evaluate T, N and M stage preoperative in patients with CRC.

In previous meta-analysis, overexpressed GLUT-1 was considered to be associated with clinical features including lymph node metastasis, T stage, higher Dukes stage and disease-free survival of CRC [4]. Overexpressed MACC1 was contribute to the transformation of malignant degree and metastatic potential of CRC, primarily for adenoma transform into Tis further transform into invasive CRC [7, 18]. Our results demonstrated a significant relationship between GLUT-1/MACC1 expression and T status (*P* = 0.006 and 0.002), differentiated degree (*P* = 0.009 and 0.014), CRC patients with high T-status and poorly differentiated tended to have higher GLUT-1/MACC1 expression levels. Our data reveals that GLUT-1 and MACC1 has a prognostic capacity for stratified tumor pathological factors (T status and differentiated degree). The study of Furudoi, A. et al. suggested that the GLUT-1 expression at the deepest invasive site was significantly with clinicopathologic features including lymph node and Duke’s stage [19]. Our finding is also consistent with Aifen Lin’s study, shown that high expression of MACC1 was correlation with higher T stage of CRC and was considered to be a valuable prognostic and risk stratification biomarker of CRC [18]. Shirahata A. et al. found that MACC1 expression was significantly correlated with peritoneal dissemination and higher TNM stage of CRC [20]. Inconsistent with previous study, the average expression of GLUT-1 and MACC1 were higher in M1 and N2 specimen but did not reach the statistical difference which may due to the limitation of small sample volume in our experiment. Moreover, our study found that the expression of MACC1 was higher than GLUT-1 in the same patient, MACC1 protein may be superior to GLUT-1 in predicting the biological characteristics of CRC.

Subsequently, our finding revealed the relationship between biomarker and radio-parameters derived from FDG PET. Our multivariate analysis shown a significantly correlation between GLUT-1 and SUVmax, MTV (*R*^*2*^ = 0.42, *P* = 0.013 and 0.004, respectively), another correlation was found between MACC1 and TLG (*R*^*2*^ = 0.372, *P* = 0.000). In most studies, SUVmax was the most commonly used parameter to evaluate FDG uptake, however, tumor volumetric parameters of FDG such as MTV and TLG provides more comprehensive information on tumor metabolism and reflect tumor biological characteristics more accurately [21]. In our study, the volumetric parameters of MTV and TLG show a power in evaluating the expression of GLUT-1 and MACC1 in CRC specimen. The mechanism of FDG uptake was acknowledged to be mainly mediated by GLUT family, and GLUT-1 was one of the most important contributors. Enhanced FDG uptake reflect the heterogeneity of glucose metabolism. The correlation between GLUT-1 and FDG uptake was found in multiple malignancies such as breast cancer [22], lung cancer [23], cervical cancer [24], pancreatic cancer [25] and colorectal cancer [12] et al. However, Ran Hong’s study show that the GLUT-1 expression was not associated with FDG uptake, the discrepancy between two studies may be caused by differences in FDG parameters, only one radio-parameter was included in Ran Hong’s research [26]. There a few studies regarded to the relationship between MACC1 and FDG uptake, the present study demonstrated a statistical correlation between MACC1 expression and TLG of CRC patients for the first time. The expression levels of MACC1 in primary CRC lesion may better reflect the character of tumor total lesion glycolysis. This correlation could be explained as the expression of MACC1 is related to tumor progression, overexpressed MACC1 enhanced the tumor Warburg effect that increasing the glucose metabolism activity of CRC [18, 27]. Consistent with Jing Liu study, demonstrates that MACC1 overexpression promotes ^18^F-FDG uptake in mice bearing NCI-N87 xenograft [28]. To investigating the relationship between biomarkers and FDG uptake will deeply understanding tumor biological behavior and lead to a clearer interpretation of PET imaging.

There were several limitations in present study. First, this study was based on a small sampling of patients who accepted ^18^F**-**FDG PET/CT scan prior to surgery, lack of patients with T1 and T2 stage. Second, the study was retrospective and we only verified the correlation between PET parameters and tumor markers in tissue level, biochemical measurement of GLUT-1 and MACC1 makers was not performed due to lack of serum samples. In future studies, larger samples with a greater number of early T stage and multiple indicators are necessary to be included to evaluating the relationship between ^18^F-FDG PET/CT parameters, tumor biomarkers and TNM stage.

## Conclusion

In summary, the present study demonstrated the feasibility of glucose metabolism parameters derived from FDG to predicting T status and M status of CRC patients noninvasively. A high level of GLUT-1 and MACC1 expression show a predict capacity for stratified tumor pathological factors (T status and differentiated degree). Moreover, overexpression of GLUT-1 and MACC1 may promote the glucose metabolism activity of CRC.

## Data Availability

The datasets used and/or analyzed during the current study are available from the corresponding author on reasonable request.

## Abbreviations

CRC: Colorectal carcinoma
CLM: Colorectal liver metastases
PET: Positron emission tomography
MACC1: Metastasis-associated in colon cancer 1
GLUT-1: Glucose transporter 1
MTV: Metabolic tumor volume
TLG: Total lesion glycolysis
